# Neuronal Nitric Oxide Synthase in Neural Stem Cells Induces Neuronal Fate Commitment via the Inhibition of Histone Deacetylase 2

**DOI:** 10.3389/fncel.2017.00066

**Published:** 2017-03-07

**Authors:** Xing Jin, Zhang-Feng Yu, Fang Chen, Guang-Xian Lu, Xin-Yuan Ding, Lin-Jun Xie, Jian-Tong Sun

**Affiliations:** ^1^Department of Pharmacy, the Affiliated Suzhou Municipal Hospital, Nanjing Medical University,Suzhou, China; ^2^Department of Critical Care Medicine, the Affiliated Suzhou Municipal Hospital, Nanjing Medical University,Suzhou, China

**Keywords:** neuronal nitric oxide synthase, histone deacetylase 2, neural stem cells, neurogenesis, differentiation

## Abstract

Active adult neurogenesis produces new functional neurons, which replace the lost ones and contribute to brain repair. Promoting neurogenesis may offer a therapeutic strategy for human diseases associated with neurodegeneration. Here, we report that endogenous neuronal nitric oxide synthase (nNOS) for neural stem cells (NSCs) or progenitors positively regulates neurogenesis. nNOS repression exhibits significantly decreased neuronal differentiation and nNOS upregulation promotes neurons production from NSCs. Using a quantitative approach, we show that instructive effect, that is instruction of NSCs to adopt a neuronal fate, contributes to the favorable effect of endogenous nNOS on neurogenesis. Furthermore, nNOS-mediated instruction of neuronal fate commitment is predominantly due to the reduction of histone deacetylase 2 (HDAC2) expression and enzymatic activity. Further investigation will be needed to test whether HDAC2 can serve as a new target for therapeutic intervention of neurodegenerative disorders.

## Introduction

It is now widely accepted that active neurogenesis persists throughout adult life in restricted brain regions of almost all mammals, including humans (Ming and Song, 2005, 2011; Bergmann et al., 2015). Adult neurogenesis is known as a process of generating new neurons from neural stem cells (NSCs), including the proliferation of NSCs or progenitors, fate determination of progenitors, and the survival, migration, maturation, and integration of new neurons (Braun and Jessberger, 2014; Jin, 2016). Failing or altered neurogenesis can cause a number of neuropsychiatric diseases, such as Alzheimer’s disease and depression (Ruan et al., 2014). Newborn neurons by replacing the lost ones may contribute to brain repair. Thus, promoting neurogenesis may represent a potential therapeutic strategy for the treatment of neuropsychiatric diseases. Many intrinsic and extrinsic factors, such as epigenetic mechanisms and neurotransmitters (Juliandi et al., 2010; Berg et al., 2013), can regulate adult neurogenesis under physiological and pathological conditions. However, the detail cellular and molecular mechanisms that guide adult neurogenesis are not completely discovered.

Nitric oxide (NO), a free gaseous neurotransmitter, is synthesized by three different isoforms of nitric oxide synthase (NOS), neuronal nitric oxide synthase (nNOS), endothelial NOS (eNOS), and inducible NOS (iNOS) (Zhou and Zhu, 2009; Berg et al., 2013). NO cannot be stored in cells and its function is dependent on new synthesis by NOS. nNOS, the NOS we focus on, is mainly expressed in cytoplasm of neurons but also been found in NSCs and astrocytes (Bredt et al., 1990; Arbones et al., 1996; Wang et al., 1999). Many studies have demonstrated that nNOS in adult brains negatively regulates neurogenesis (Packer et al., 2003; Moreno-Lopez et al., 2004; Luo et al., 2014). We hold the opinion that the negative effect of nNOS on neurogenesis *in vivo* is produced by neuron-derived nNOS because of main expression of nNOS in neurons but little in NSCs and astrocytes. Our preliminary data suggest that NSC-derived nNOS opposite to neuron-derived nNOS is required for neuronal differentiation (Luo et al., 2010). However, we still do not know whether NSC-derived nNOS contributes to neuronal fate commitment or the favorable effect on neuronal differentiation is just due to the proliferation of NSCs or reduced apoptosis of new neurons. Furthermore, the detail mechanisms underlying the role of endogenous nNOS in neurogenesis remain unclear.

Histone deacetylases (HDACs) have been implicated in the epigenetic regulation of neurogenesis by chromatin structure modulation (Ma et al., 2010; Jobe et al., 2012). Mammalian HDACs including HDAC1∼11 and SIRT1∼7 have been classified into three classes (Marks et al., 2003). Among HDACs, HDAC2 grouped in class I is selectively expressed in neurons but not in glial lineage cells during NSC differentiation (Juliandi et al., 2010). HDAC2 has been shown to negatively regulate synaptic plasticity and affect neurological functions such as regulating learning and memory (Guan et al., 2009; Kramer, 2009; Nott et al., 2015). Here, we show that HDAC2 negatively regulates neuronal fate commitment and mediates the instructive effect of endogenous nNOS on fate commitment by NSCs to a neuronal lineage.

## Materials and Methods

### Animals

Homozygous nNOS-deficient mice (B6; 129S4-Nos1^tm1Plh^, nNOS^-/-^) and their wild-type controls of similar genetic background (B6129SF2, WT) (both from Jackson Laboratories) were maintained in Model Animal Research Center of Nanjing University. Adult male (2-month-old) and embryonic ICR mice (Shanghai Silaike Laboratory Animal) were also used in this study. All experimental protocols using animals were approved by the Institutional Animal Care and Use Committee of Nanjing Medical University.

### Cell Culture

Embryonic NSCs were isolated from embryonic day 14 (E14) mouse cortex as we previously described (Luo et al., 2010), with minor modifications. Cells were floating cultured in proliferation medium, DMEM/F12 medium (1:1; Invitrogen) containing 20 ng/ml basic fibroblast growth factor (bFGF; Sigma-Aldrich), 20 ng/ml epidermal growth factor (EGF; Sigma-Aldrich), and 2% B27 supplement (Invitrogen), and passaged every 4–6 days. Embryonic NSCs of the second to fifth passage were used in this study.

Adult NSCs were isolated and cultured as previously described (Luo et al., 2010), with some modifications. In brief, the dentate gyri of 2-month-old male mice (10 mice were used in each primary isolation experiment) were dissected and digested with 0.125% trypsin (Invitrogen) at 37°C for 15 min. Then the digestion was terminated with DMEM/F12 medium containing 10% fetal bovine serum (FBS; TBD), and serum-free DMEM/F12 medium was used to wash remanent FBS. Finally, cells were centrifuged and resuspended in proliferation medium, seeded at 1 × 10^6^ cells/ml, and maintained at 37°C with 5% CO_2_. Adult NSCs of the second to fourth passage were used in this study.

### NSC Differentiation

Monolayer-cultured NSCs were allowed to differentiate in differentiation medium (DMEM/F12 medium containing 2% B27 and 0.5% FBS). Cells underwent different treatments and were fixed at different time points for immunofluorescence label.

For HDAC2 expression and enzymatic activity assay, neurospheres were planted in differentiation medium in cell culture dishes coated with polyornithine (10 μg/ml; Sigma-Aldrich), and were immediately treated with vehicle or 100 μM *N*^5^-(1-imino-3-butenyl)-L-ornithine (L-VNIO; Alexis Biochemicals) for 24 h. Then the protein was extracted.

### Cell Proliferation Assay

Cell proliferation was assessed by cell counting for the neurosphere-cultured NSCs and by bromodeoxyuridine (BrdU) incorporation for the monolayer-cultured NSCs. For cell counting, single-cell suspension was seeded at 2 × 10^4^ cells/ml. Four days later, new-generated neurospheres were dissociated to single cells and the number of cells was counted on a hemocytometer. Data were normalized to the percentage of control.

For BrdU incorporation, NSCs were plated on coverslips coated with polyornithine and laminin (5 μg/ml; Invitrogen) and cultured as a monolayer. The cells were treated with 2.5 μM BrdU (Sigma-Aldrich) during the later 2 days of the 4-day differentiation, or 10 μM BrdU during the last 2 h of the 24-h proliferation culture, or 10 μM BrdU for 24 h for identification of cultured adult NSCs. Then the cultures were fixed in phosphate-buffered solution (PBS) containing 4% paraformaldehyde for 15 min at room temperature and BrdU^+^ cells were visualized by immunofluorescence label.

### Immunofluorescence

Fixed cultures were blocked in PBS with 3% goat serum, 0.3% Triton X-100, and 0.1% bovine serum albumin at room temperature for 1 h, followed by incubation with primary antibody at 4°C overnight and then secondary antibody for 2 h at room temperature. The primary antibodies used were as follows: mouse anti-nestin (1:100; sc-33677; Santa Cruz Biotechnology), mouse anti-β-III-Tubulin (1:200; MAB1637; Millipore Bioscience Research Reagents) or mouse anti-glial fibrillary acidic protein (GFAP; 1:1000; MAB360; Millipore Bioscience Research Reagents). The fluorescent secondary antibody used was goat anti-mouse DyLight488 (1:400; 515-485-062; Jackson ImmunoReasch). Finally, Hoechst 33258 (Sigma-Aldrich) was used to label the nuclei.

For BrdU immunofluorescence, fixed membranes were first ruptured by 0.2% Triton X-100. Then, the cells were denatured in 2 M HCl (20°C for 10 min) and rinsed in 0.1 M boric acid (pH 8.5) for 10 min before blocking. The next steps were the same to those mentioned above in immunofluorescence. The primary antibody was mouse anti-BrdU (1:1000; MAB4072; Millipore Bioscience Research Reagents), and the secondary antibody was goat anti-mouse Cy3 (1:200; 115-165-003; Jackson ImmunoReasch).

All these images of immunostained cells were captured with a fluorescence microscope (Axio Imager; Zeiss). The percentages of neurons, astrocytes and BrdU-labeled dividing cells were calculated in 40 high-power fields systematically across the coverslip. The cells were counted using Image-Pro (Media Cybernetics).

### Cell Apoptosis and Necrosis Assay

Cell apoptosis and necrosis assay kit (Beyotime) was used to detect dead cells during NSC differentiation. Living cultures were stained with propidium iodide (PI) which stains dead cells and Hoechst 33342 which stains live and dead cells for 20 min at 37°C and imaged with a fluorescence microscope at 40×.

### Quantitative Analysis of Neurogenesis from NSCs

A mathematical description of neurogenesis was used to analyze the effect of nNOS from NSCs on neuronal fate as previously described (Song et al., 2002; Hsieh et al., 2004a), with some modifications. PI_j_ is the number of dead cells present at the start of the j day in culture, N_j_ is the total number of cells present at that time, δ_j_ is the death rate of all cells. δ_j_ is given by the equation δ_j_ N_j_ = PI_j+1_ – PI_j_. In addition, n_j+1_ is the total number of neurons present at the start of the (j+1) day, and it is given by the equation n_j+1_ = n_j_ + β_j_ (N_j_-n_j_) – δ_j_^n^n_j_, where β_j_ is the conversion rate of progenitors to neurons for that day and δ_j_^n^ is the death rate of neurons. The second term on the right is the number of neurons generated by conversion of neural progenitors, and the third term is the number of dead neurons. If δ_j_^n^n_j_ = δ_j_ N_j_, that is all dead cells are neurons, we can obtain β_j_.

### Western Blot Analysis

Western bolt analysis was performed as described previously (Luo et al., 2007). The primary antibodies were as follows: mouse anti-nNOS (1:600; 610308; BD Biosciences), mouse anti-HDAC2 (1:2000; ab51832; Abcam) or mouse anti-GAPDH (1:2500; KC-5G4; KangChen Bio-tech). Appropriate horseradish peroxidase-linked secondary antibodies were used for detection by enhanced chemiluminescence (Pierce).

### HDAC2 Activity Assay

Histone deacetylase activity was assessed using a fluorometric HDAC assay kit (EMD Millipore). For HDAC2-specific activity, immunoprecipitation with the specific antibody was performed before the assay as described previously (Guan et al., 2009; Luo et al., 2014). Briefly, 0.5 μg of mouse anti-HDAC2 antibody (Abcam) was added to the cellular lysates and incubated for 12 h at 4°C. Then 20 μl of protein G-Agarose (Sigma-Aldrich) was added and incubated on a tube rotator for 4 h at 4°C. Beads were centrifuged at 2500 × *g* and washed five times in immunoprecipitation buffer (50 mM Tris-HCl, 150 mM NaCl, 5 mM EDTA, 0.5% NP40, pH 8.0, supplemented with 1 mM PMSF). Finally, HDAC assay substrate was added to the beads and incubated at 30°C for 45 min, after which the reaction was stopped and fluorescence was measured according to the instructions of HDAC assay kit.

### Recombinant Virus Infection

#### AD-HDAC2

The recombinant adenovirus, AD-HDAC2 or its control AD-Null, was generated by GeneChem as we previously reported (Luo et al., 2014). AD-HDAC2 overexpresses mouse HDAC2 protein. Cultured NSCs were infected with AD-Null or AD-HDAC2 containing 1.5 × 10^9^ plaque-forming units/ml for 12 h *in vitro* (multiplicity of infection = 3). The adenovirus-infected NSCs then proliferated or differentiated for 4 days. The procedures concerning recombinant adenovirus were performed following National Institutes of Health guidelines.

#### LV-HDAC2 shRNA

HDAC2 shRNA lentiviral particles (LV-HDAC2 shRNA; Santa Cruz Biotechnology) are recommended for the inhibition of HDAC2 expression in mouse cells. LV-Control shRNA (Santa Cruz Biotechnology) or LV-HDAC2 shRNA containing 1.0 × 10^6^ infectious units/200 μl/vial was infected into cultured NSCs at 10 μl/well (24-well plate) when NSCs were passaged at 2 × 10^4^ cells/cm^2^. Twelve hours later, the medium was fully changed with fresh proliferation medium. The lentivirus-infected NSCs proliferated for 4 days and were passaged for experiments. The procedures concerning recombinant lentivirus were performed following National Institutes of Health guidelines.

### Statistical Analysis

Comparisons were made with one-way ANOVA followed by Scheffe’s *post hoc* test. Data were presented as mean ± SEM. Differences were considered significant when *p* < 0.05.

## Results

### Endogenous nNOS Is Required for Neuronal Differentiation of Embryonic NSCs

**FIGURE 1:**
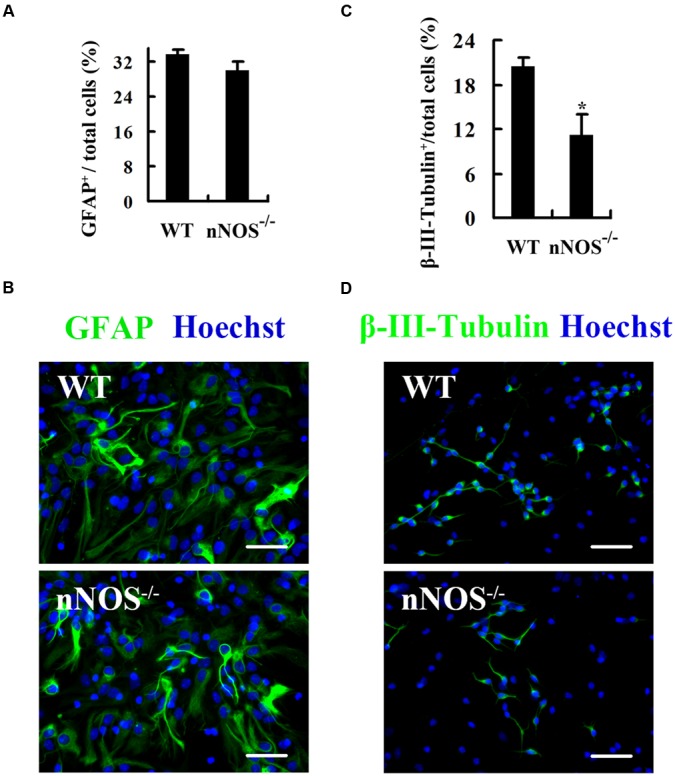
**Endogenous nNOS is required for neuronal differentiation of embryonic NSCs.** The monolayer-cultured NSCs from nNOS^-/-^ and WT embryonic mice differentiated for 4 days and then were fixed for stain. **(A)** Statistical graph showing the ratio of GFAP^+^ astrocytes. **(B)** Representatives of GFAP-labeled cells. **(C)** Statistical graph showing the ratio of β-III-Tubulin^+^ neurons. **(D)** Representatives of β-III-Tubulin^+^ cells. Nuclei were counterstained with Hoechst 33258. Scale bars = 50 μm. Data are mean ± SEM (*n* = 3); ^∗^*p*<0.05 as compared with WT. GFAP, glial fibrillary acidic protein; nNOS^-/-^, gene knockout of neuronal nitric oxide synthase; WT, wild-type.

To investigate the role of endogenous nNOS in neuronal differentiation, we cultured embryonic NSCs from nNOS^-/-^ and WT mice. Monolayer-cultured NSCs proliferated for 24 h and then were allowed to differentiate for 4 days. We found that although nNOS gene knockout had much weak effect on the percentage of astrocytes (GFAP^+^; **Figures 1A,B**), it substantially reduced neuronal differentiation (β-III-Tubulin^+^) (WT 20.39 ± 1.20% vs. nNOS^-/-^ 11.21 ± 2.69%, *p* < 0.05; **Figures 1C,D**), indicating that nNOS in NSCs is necessary for neuronal differentiation *in vitro*.

### Repression of nNOS Negatively Regulates Neuronal Fate Commitment

**FIGURE 2:**
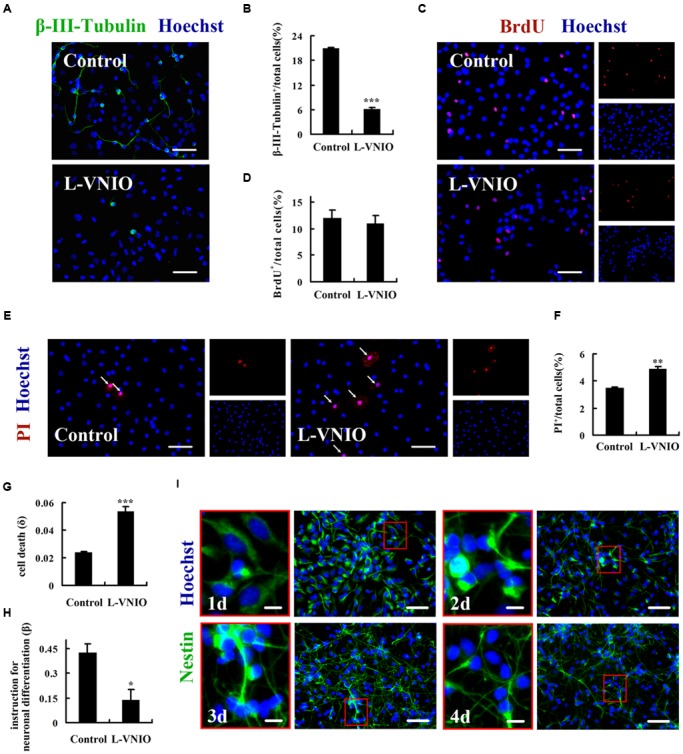
**Repression of nNOS negatively regulates neuronal fate commitment.** Monolayer-cultured embryonic NSCs differentiated for 4 days and were incubated with L-VNIO (100 μM) or vehicle during the later 2 days of differentiation. **(A,B)** L-VNIO inhibits neuronal differentiation. **(A)** Representatives of β-III-Tubulin^+^ neurons. **(B)** Statistical graph showing the ratio of β-III-Tubulin^+^ neurons. **(C,D)** L-VNIO has no effect on cell proliferation during NSCs differentiation. BrdU (2.5 μM) was added during the later 2 days of differentiation to label the dividing cells. **(C)** Representatives of BrdU-labeled cells. **(D)** Statistical graph showing the ratio of BrdU^+^ cells. **(E,F)** L-VNIO induces cell apoptosis. Live cultures were stained with PI which stains dead cells and Hoechst 33342 which stains live and dead cells. **(E)** Representatives of PI^+^ nuclei (arrows). **(F)** Statistical graph showing the ratio of PI^+^ nuclei. **(G)** L-VNIO increases the rate of cell death (δ_4_). **(H)** L-VNIO decreases the rate of conversion of progenitors to neurons (β_4_) even if all dead cells are neurons. **(I)** There are nestin^+^ progenitor cells throughout the differentiation stages. Cells were fixed and stained for nestin at days 1, 2, 3, 4 after differentiation respectively. Data shown are mean ± SEM from three to five independent experiments in parallel cultures; ^∗^*p* < 0.05, ^∗∗^*p* < 0.01, ^∗∗∗^*p* < 0.001 vs. control. Scale bars = 50 μm [**A,D,E,I** (right)], 10 μm [**I** (left)]. BrdU, bromodeoxyuridine; L-VNIO, *N*^5^-(1-imino-3-butenyl)-L-ornithine; PI, propidium iodide.

The reduction of neuronal differentiation by nNOS gene knockout could be due to some combination of decreased proliferation of NSCs or progenitors, attenuated neuronal survival, and/or inhibition of fate commitment by NSCs to a neuronal lineage. First, we examined whether a decrease in cell proliferation or survival might explain the reduced number of neurons in nNOS-repression cultures. L-VNIO, a highly selective inhibitor of nNOS (Babu and Griffith, 1998), was used to inhibit nNOS enzymatic activity. Cultures were treated with 100 μM L-VNIO or vehicle for the later 2 days during the 4-day differentiation of monolayer-cultured embryonic NSCs, immediately followed by fixation and staining with antibody against β-III-Tubulin. Consistent with results of nNOS gene knockout, we observed that L-VNIO did not affect glial differentiation (Supplementary Figure S1), but remarkably reduced the percentage of neurons (β-III-Tubulin^+^) at day 4 after differentiation (Control 20.83 ± 0.31% vs. L-VNIO 6.23 ± 0.40%, *p* < 0.001; **Figures 2A,B**). The reduction of neuronal differentiation was not due to decreased proliferation of progenitors, since L-VNIO did not inhibit cell proliferation (**Figures 2C,D**). However, PI staining revealed that L-VNIO significantly increased the apoptotic ratio of cells (PI^+^) (Control 3.48 ± 0.07% vs. L-VNIO 4.91 ± 0.15%, *p* < 0.01; **Figures 2E,F**). Thus, cell apoptosis may contribute to the effect of L-VNIO on decreased neurogenesis.

Does the repression of nNOS also inhibit progenitors to adopt a neuronal fate? To address this question, we used a mathematical description of neurogenesis, which is developed by Song et al. (2002). During the process of quantitative description, we defined δ_j_ as the death rate of all cells for the jth day in culture, and β_j_ as the conversion rate of progenitors (Tuj1^-^) to neurons (Tuj1^+^) during that day. The death rate, δ_j_, was measured by using PI dye in live cultures and was given by the equation δ_j_ N_j_ = PI_j+1_ – PI_j_. PI_j_ is the number of dead cells present at the start of the j day in culture and N_j_ is the total number of cells present at that time. Consistent with results of **Figures 2E,F**, we found that the death rate (δ_4_) is significantly higher in L-VNIO-treated cultures (Control 0.024 ± 0.0007 vs. L-VNIO 0.054 ± 0.0033, *p* < 0.001; **Figure 2G**). The rate of conversion of progenitors to neurons, β_j_, was given by the equation n _j+1_ = n_j_ + β_j_ (N_j_-n_j_) – δ_j_^n^n_j_. n_j_ is the total number of neurons present at the start of the j day and δ_j_^n^ is the death rate of neurons for the jth day. However, we could not determine what fractions of the dead cells were neurons. If no neurons die, we can say L-VNIO decreases the rate of neuronal fate commitment of NSCs. If all cells that die are neurons, that is, δ_j_^n^n_j_ = δ_j_ N_j_, we can obtain β_j_. As shown in **Figure 2H**, L-VNIO leads to notably lower value of β_j_ (*j* = 4) (Control 0.426 ± 0.05 vs. L-VNIO 0.136 ± 0.06, *p* < 0.05). In addition, nestin labeling experiments showed that there are still neural progenitor cells at day 4 after differentiation (**Figure 2I**). Taken together, these results demonstrate that repression of nNOS negatively regulates neuronal fate commitment of embryonic NSCs.

### Upregulation of Endogenous nNOS Promotes Neuronal Differentiation

**FIGURE 3:**
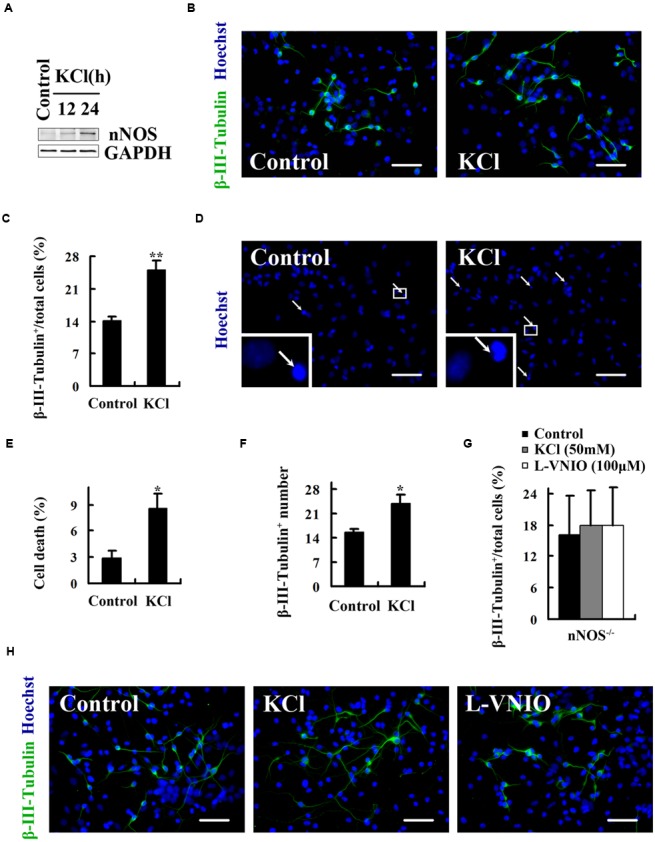
**Upregulation of endogenous nNOS promotes neuronal differentiation. 50 mM KCl was treated during the first 12 (A)** or 24 **(A–H)** hours, and 100 μM L-VNIO for the later 2 days of embryonic NSCs differentiation. **(A)** KCl increases nNOS expression in neural progenitor cells at day 1 after differentiation. **(B,C)** KCl markedly increases the percentage of neurons at day 4 after differentiation. **(B)** Representatives of β-III-Tubulin^+^ neurons. **(C)** Statistical graph from data in **(B)**. **(D,E)** KCl induces cell apoptosis. Cell death was assessed by staining with Hoechst 33258 to visualize fragmented nuclei at day 4 after differentiation. **(D)** It shows example images in Hoechst 33258 fluorescence. Arrows mark dying cells. **(E)** Statistical graph from data in **(D)**. **(F)** Cultures treated with KCl exhibit notably increased number of neurons at day 4 after differentiation. **(G,H)** The effect of KCl and L-VNIO on neuronal differentiation is abolished when nNOS is knocked out. **(G)** Statistical graph showing the ratio of neurons. **(H)** Representatives of β-III-Tubulin^+^ neurons. Scale bars = 50 μm. Data are mean ± SEM (*n* = 3); ^∗^*p* < 0.05, ^∗∗^*p* < 0.01 as compared with control. GAPDH, glyceraldehyde phosphate dehydrogenase; L-VNIO, *N*^5^-(1-imino-3-butenyl)-L-ornithine; nNOS, neuronal nitric oxide synthase.

To examine the effect of nNOS upregulation on neuronal differentiation, we incubated the cultures with 50 mM KCl during the first 12 or 24 h of NSCs differentiation. Sasaki et al. (2000) demonstrated that 50 mM KCl induces nNOS expression in cortical neurons. Our results showed that 50 mM KCl for 24 h clearly increased nNOS expression at day 1 after differentiation (**Figure 3A**), when almost all cells are nestin^+^ neural progenitors (**Figure 2I**). Treatment with 50 mM KCl for the first 24 h significantly promoted neuronal differentiation at day 4 after differentiation. The ratio of β-III-Tubulin^+^ neurons in KCl-treated cultures was 24.95 ± 2.07%, compared with 14.07 ± 1.03% in control (**Figures 3B,C**, *p* < 0.01). Moreover, 50 mM KCl markedly induced cell apoptosis (**Figures 3D,E**). The apoptotic effect was not mediated by nNOS since nNOS gene knockout did not abolish the effect (Supplementary Figure S2). If all dead cells are astrocytes, the data of increased neuronal ratio in KCl-treated cultures might be superficial. We then counted the number of β-III-Tubulin^+^ cells, which was proved to be much higher in KCl-treated cultures (**Figure 3F**). To confirm that nNOS mediates the effect of KCl and L-VNIO on neuronal differentiation, we treated nNOS^-/-^ cells with 50 mM KCl for the first 24 h or 100 μM L-VNIO for the later 2 days of 4-day differentiation. As shown in **Figures 3G,H**, nNOS gene knockout abolished the effect of KCl and L-VNIO on neuronal differentiation. These findings collectively indicate that upregulation of endogenous nNOS promotes neuronal differentiation.

### HDAC2 Mediates the Role of nNOS in Regulating the Fate of Embryonic NSCs

**FIGURE 4:**
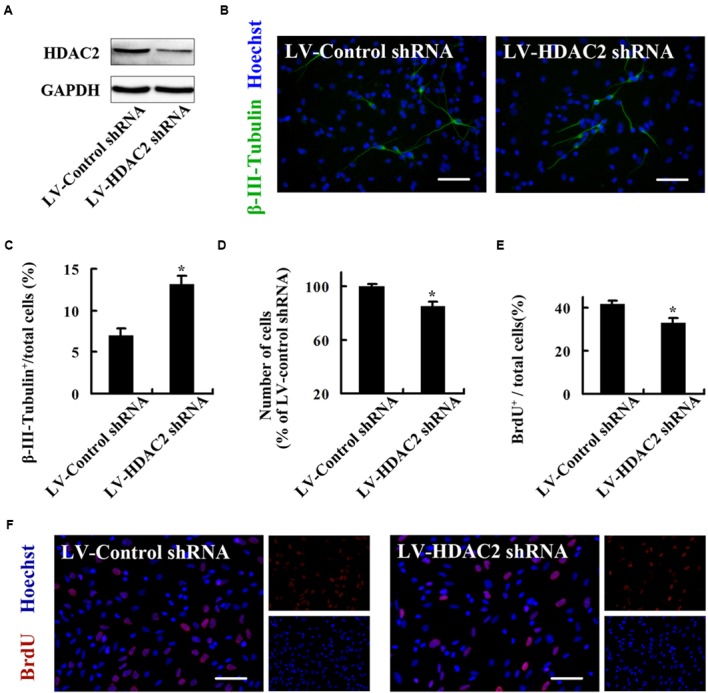
**HDAC2 down-regulation instructs embryonic NSCs to exit from the cell cycle and adopt a neuronal fate. (A)** Immunoblots showing HDAC2 levels in the NSCs infected by LV-HDAC2 shRNA or LV-Control shRNA at day 4 after infection. **(B,C)** NSCs infected by LV-HDAC2 shRNA differentiate into notably more neurons after differentiation for 4 days. **(B)** Representatives of β-III-Tubulin^+^ neurons. **(C)** Statistical graph from data in **(B)**. **(D)** LV-HDAC2 shRNA inhibits NSCs proliferation, quantified by cell counting. **(E,F)** LV-HDAC2 shRNA inhibits NSCs proliferation, quantified by BrdU incorporation. BrdU (10 μM) was introduced during the last 2 h of the 24-h proliferation culture. **(E)** Statistical graph showing the percentage of BrdU^+^ cells. **(F)** Representatives of BrdU-labeled cells of the monolayer-cultured NSCs. Scale bars = 50 μm. Data shown are mean values ± SEM from three experiments in parallel cultures; ^∗^*p* < 0.05 vs. LV-Control shRNA. BrdU, bromodeoxyuridine; GAPDH, glyceraldehyde phosphate dehydrogenase; HDAC2, histone deacetylase 2; LV-Control shRNA, lentiviral vector containing control shRNA; LV-HDAC2 shRNA, lentiviral vector containing shRNA of HDAC2.

**FIGURE 5:**
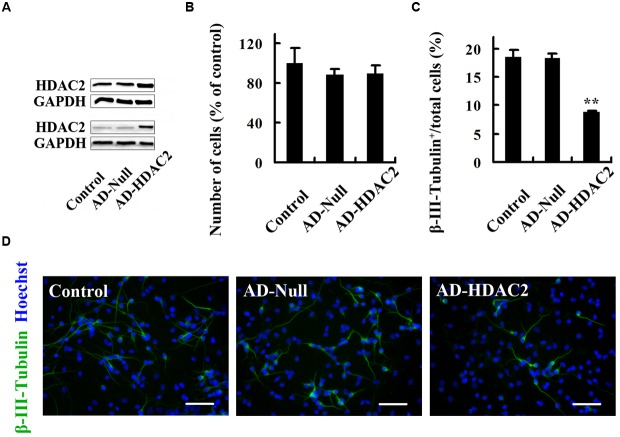
**HDAC2 up-expression has no effect on NSC proliferation but reduces neuronal differentiation. (A)** Immunoblots showing HDAC2 levels at day 4 after proliferation (top) or differentiation (bottom) of AD-Null- or AD-HDAC2-infected NSCs. **(B)** Statistical graph showing the number of cells of neurospheres at day 4 after proliferation of AD-Null- or AD-HDAC2-infected NSCs. **(C,D)** AD-HDAC2 inhibits neuronal differentiation. AD-Null- or AD-HDAC2-infected NSCs differentiated for 4 days and then were fixed for β-III-Tubulin staining. **(C)** Statistical graph showing the ratio of β-III-Tubulin^+^ neurons. **(D)** Example images of β-III-Tubulin^+^ neurons. Scale bars = 50 μm. Data shown are mean values ± SEM from three experiments in parallel cultures; ^∗∗^*p* < 0.01 vs. AD-Null. AD-HDAC2, adenovirus vector selectively expressing HDAC2; GAPDH, glyceraldehyde phosphate dehydrogenase; HDAC2, histone deacetylase 2.

To explore the molecular mechanisms underlying the regulation of NSC fate by nNOS, we investigated the role of HDAC2. To regulate HDAC2 specifically, we generated a lentiviral vector that contains shRNA of HDAC2 and named it LV-HDAC2 shRNA. LV-HDAC2 shRNA effectively infected NSCs and significantly decreased HDAC2 expression (**Figure 4A**). LV-HDAC2 shRNA-infected NSCs gave rise to significantly increased numbers of neurons (**Figures 4B,C**) but exhibited a decrease in their own proliferation (**Figures 4D–F**), suggesting that HDAC2 down-regulation instructs NSCs to exit from the cell cycle and adopt a neuronal fate. To further confirm the effect of HDAC2 in NSCs on neurogenesis, we infected NSCs with an adenovirus vector selectively expressing HDAC2 (AD-HDAC2) for 12 h, then AD-HDAC2 increased HDAC2 expression at day 4 after proliferation (**Figure 5A**, top) or differentiation (**Figure 5A**, bottom). Unexpectedly, AD-HDAC2 had no effect on NSC proliferation (**Figure 5B**), but significantly decreased neuronal differentiation (**Figures 5C,D**). Both LV-HDAC2 shRNA and AD-HDAC2 did not affect cell apoptosis (data not shown). Thus, HDAC2 negatively regulates neuronal fate commitment of embryonic NSCs.

**FIGURE 6:**
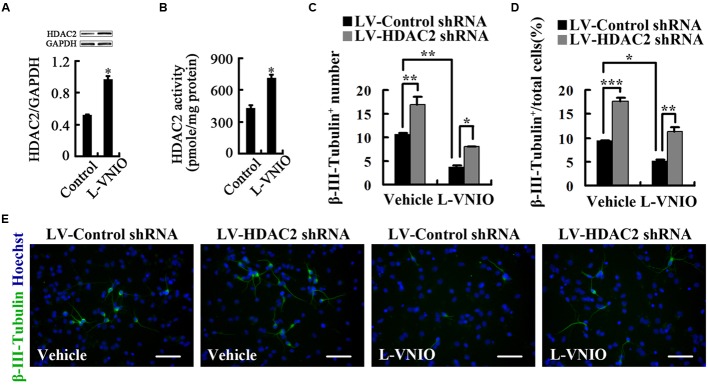
**Repression of nNOS negatively regulates differentiation of embryonic NSCs into neurons by upregulation of HDAC2. (A,B)** HDAC2 levels **(A)** and enzymatic activity **(B)** in cultures treated with 100 μM L-VNIO or vehicle for the first 24 h during differentiation. **(C–E)** HDAC2 down-regulation rescues L-VNIO-induced neuronal differentiation reduction. 100 μM L-VNIO or vehicle was treated for the later 2 days during the 4-day differentiation of LV-HDAC2 shRNA- or LV-Control shRNA-infected NSCs. **(C)** Bar graph showing the number of newborn neurons. **(D)** Bar graph showing the percentage of newborn neurons. **(E)** Representatives of β-III-Tubulin^+^ neurons. Scale bars = 50 μm. Data are mean ± SEM (*n* = 3); ^∗^*p* < 0.05, ^∗∗^*p* < 0.01, ^∗∗∗^*p* < 0.001. GAPDH, glyceraldehyde phosphate dehydrogenase; HDAC2, histone deacetylase 2; LV-Control shRNA, lentiviral vector containing control shRNA; LV-HDAC2 shRNA, lentiviral vector containing shRNA of HDAC2; L-VNIO, *N*^5^-(1-imino-3-butenyl)-L-ornithine.

Next, we asked whether nNOS could regulate HDAC2, thereby affecting the fate of NSCs. We planted neurospheres in polyornithine-coated cell culture dishes in differentiation medium and immediately treated cells with 100 μM L-VNIO or vehicle for 24 h. We found that L-VNIO significantly increased HDAC2 expression and enzymatic activity at day 1 after differentiation (**Figures 6A,B**). To determine whether HDAC2 upregulation mediates the role of L-VNIO in inhibition of fate commitment by NSCs to a neuronal lineage, we treated cultures with 100 μM L-VNIO or vehicle for the later 2 days during the 4-day differentiation of LV-HDAC2 shRNA- or LV-Control shRNA-infected NSCs. HDAC2 down-regulation rescued L-VNIO-induced neuronal differentiation reduction (**Figures 6C–E**). Together, nNOS repression negatively regulates neuronal fate commitment by upregulation of HDAC2 in progenitor cells.

### HDAC2 Mediates the Role of nNOS in Regulating the Fate of Adult NSCs

**FIGURE 7:**
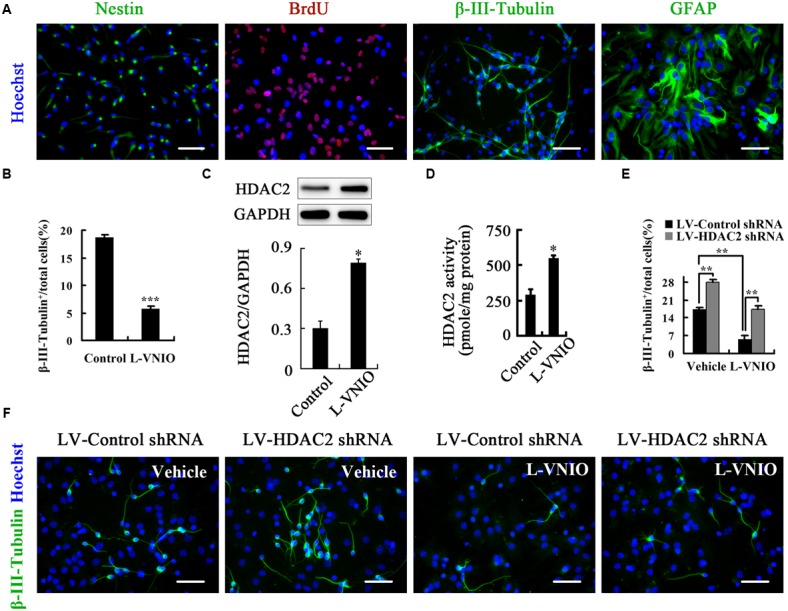
**HDAC2 mediates the role of nNOS in regulating the fate of adult NSCs. (A)** Identification of cultured adult NSCs. Single-cell suspensions were seeded on polyornithine/laminin-coated coverslips, cultured as a monolayer for 24 h, and then fixed for nestin staining. At least 92% cells were nestin^+^ NSCs. In addition, cells were monolayer-cultured in the presence of 10 μM BrdU for 24 h and fixed for stain, and most cells were BrdU^+^ labeled. These cells could differentiate into β-III-Tubulin^+^ neurons and GFAP^+^ astrocytes after differentiation for 4 days. **(B)** Monolayer-cultured adult NSCs treated with 100 μM L-VNIO during the later 2 days of 4-day differentiation exhibit a marked decrease of neuronal differentiation. Immunoblots showing HDAC2 levels **(C)** and bar graph showing HDAC2 activity **(D)** in cultures treated with 100 μM L-VNIO or vehicle for the first 24 h during differentiation. **(E,F)** HDAC2 down-regulation rescues L-VNIO-induced neuronal differentiation reduction. 100 μM L-VNIO or vehicle was treated for the later 2 days during the 4-day differentiation of LV-HDAC2 shRNA- or LV-Control shRNA-infected adult NSCs. **(E)** Bar graph showing the percentage of newborn neurons. **(F)** Representatives of β-III-Tubulin^+^ neurons. Scale bars = 50 μm. Data are mean ± SEM (*n* = 3); ^∗^*p* < 0.05, ^∗∗^*p* < 0.01, ^∗∗∗^*p* < 0.001. BrdU, bromodeoxyuridine; GAPDH, glyceraldehyde phosphate dehydrogenase; GFAP, glial fibrillary acidic protein; HDAC2, histone deacetylase 2; LV-Control shRNA, lentiviral vector containing control shRNA; LV-HDAC2 shRNA, lentiviral vector containing shRNA of HDAC2; L-VNIO, *N*^5^-(1-imino-3-butenyl)-L-ornithine.

To determine whether HDAC2 is a mediator for the regulation of NSC fate by nNOS, we cultured adult NSCs isolated from the hippocampus of 2-month-old male mice. These adult NSCs express nestin--- a marker of neural stem/progenitor cells, actively proliferate and differentiate into neurons (β-III-Tubulin^+^) and astrocytes (GFAP^+^) (**Figure 7A**). L-VNIO (100 μM) was added to the cultures at the start of day 3 during differentiation of monolayer-cultured adult NSCs. After 48 h, L-VNIO significantly inhibited neuronal differentiation (**Figure 7B**). Furthermore, 100 μM L-VNIO added for the first 24 h during differentiation dramatically increased HDAC2 expression and enzymatic activity at day 1 after differentiation (**Figures 7C,D**). LV-HDAC2 shRNA-infected adult NSCs differentiated into more neurons, and the negative effect of L-VNIO on neuronal differentiation was abolished in LV-HDAC2 shRNA-infected adult NSCs (**Figures 7E,F**), suggesting that HDAC2 upregulation mediates the effect of nNOS repression on inhibiting differentiation of adult NSCs into neurons.

## Discussion

In this study, we have examined the role of endogenous nNOS in neurogenesis by nNOS gene knockout and pharmacological intervention. We demonstrated that endogenous nNOS promotes neuronal differentiation. Using a quantitative approach, we showed that nNOS in neural progenitors instructs neuronal fate commitment. We also explored the molecular mechanism underlying and established that the changes of HDAC2 expression and enzymatic activity may be critical.

Neuronal nitric oxide synthase, mainly responsible for NO production in central nervous system, has been implicated in the regulation of various physiological and pathological functions, such as neurogenesis and neurodegeneration (Zhang and Snyder, 1995; Boehning and Snyder, 2003). In our previous studies, we found that NSC-derived nNOS is located in nucleus and expressed at a much lower level than that in neurons (Luo et al., 2010). The nuclei localization and lower expression of nNOS in NSCs induce that NO hardly diffuses to the outside of NSCs and may act by nuclear signaling molecule. However, neuron-derived NO can diffuse into the cytoplasms of neighboring cells and probably acts via cytoplasmic signaling molecule. The differences raise a possibility that different sources of NO play different roles. We recently proved that neuron-derived NO negatively regulates neurogenesis under physiological and pathological conditions (Luo et al., 2010, 2014). In the present study, we found that NSCs lacking nNOS gene differentiated into fewer neurons, suggesting a positive role of endogenous nNOS in neurogenesis. Did the nNOS instruct progenitors to adopt a neuronal fate? Using a quantitative approach, we separated instructive effects from other possibilities, including cell proliferation and survival. Our results showed that nNOS in progenitors was required for neuronal fate commitment and cell survival, but not required for cell proliferation probably due to the much low rate of proliferation during the later 2 days of differentiation (Supplementary Figure S3).

A main part of our research is to explore the mechanism underlying the role of endogenous nNOS in neurogenesis. HDAC inhibition mediated by non-selective inhibitors has been shown to promote neurogenesis (Hsieh et al., 2004b; Balasubramaniyan et al., 2006). However, non-selective blockade of HDACs has been reported to cause a range of untoward effects (Bruserud et al., 2007). Thus, one specific HDAC family member involved in neurogenesis may provide a safer strategy. Here we transfected NSCs with LV-HDAC2 shRNA or AD-HDAC2, and confirmed that HDAC2 negatively regulates neuronal fate commitment of NSCs. As a nuclear signaling molecule (Walkinshaw et al., 2008), HDAC2 may mediate the role of endogenous nNOS. Our findings proved the notion and indicated that nNOS-mediated instruction of fate commitment by NSCs to a neuronal lineage is predominantly due to the reduction of HDAC2 expression and enzymatic activity. However, HDAC2 impossibly mediates nNOS-required cell survival because both LV-HDAC2 shRNA and AD-HDAC2 do not induce cell apoptosis. This is why nNOS repression also decreases neuronal ratio in LV-HDAC2 shRNA-infected cultures (**Figures 6C,D**, **7E**). Not only do these results reveal important functions of HDAC2 in nNOS-required neurogenesis, they also suggest that HDAC2 could be a primary target for therapeutic intervention of neurodegenerative disorders since selective increase of nNOS in NSCs but not in neurons is a great challenge.

## Author Contributions

XJ: conception and design, data analysis and interpretation, manuscript writing, final approval of manuscript; Z-FY and FC: conception and design, collection and assembly of data, data analysis and interpretation, final approval of manuscript. G-XL, X-YD, and L-JX: collection and assembly of data, final approval of manuscript. J-TS: conception and design, data analysis and interpretation, manuscript writing, final approval of manuscript.

## Conflict of Interest Statement

The authors declare that the research was conducted in the absence of any commercial or financial relationships that could be construed as a potential conflict of interest.
