# Supplementary material for: Neuronal Nitric Oxide Synthase in Neural Stem Cells Induces Neuronal Fate Commitment via the Inhibition of Histone Deacetylase 2
Source: Front Cell Neurosci. 2017 Mar 7;11:66. doi: 10.3389/fncel.2017.00066 (PMC5339248; doi:10.3389/fncel.2017.00066)
Supplement: Supplementary file 1 [file Data_Sheet_1.DOC]

Supplementary Material

**Neuronal Nitric Oxide Synthase in Neural Stem Cells Induces Neuronal Fate Commitment via the Inhibition of Histone Deacetylase 2**

**Xing Jin^┼^, Zhang-Feng Yu^┼^, Fang Chen^┼^, Guang-Xian Lu, Xin-Yuan Ding, Lin-Jun Xie, Jian-Tong Sun^*^**

***Correspondence:** Jian-Tong Sun: [18915591290@163.com](mailto:18915591290@163.com)

**^┼^**These authors have contributed equally to this work.

**Supplementary Figures**

**
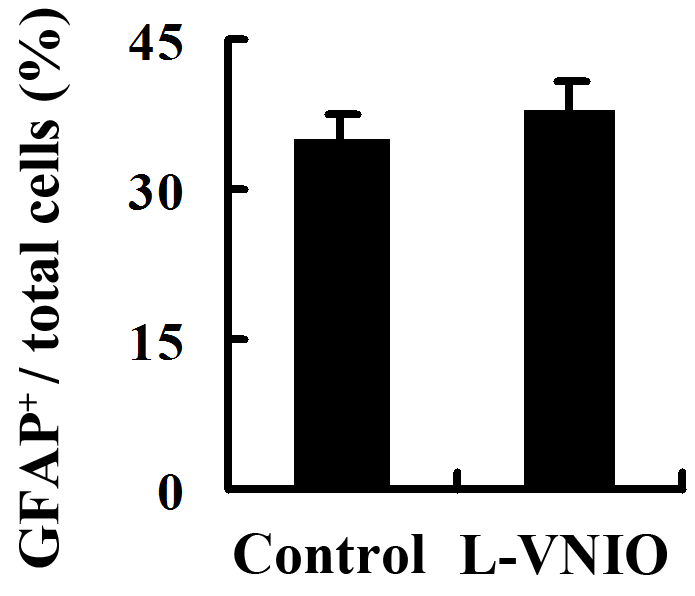
**

**Supplementary Figure 1.** L-VNIO has no effect on glial differentiation. Monolayer-cultured embryonic NSCs differentiated for 4 days and were incubated with L-VNIO (100μM) or vehicle during the later two days of differentiation. Data are means ± SEM (n=3). Abbreviations: GFAP, glial fibrillary acidic protein; L-VNIO, N5-(1-imino-3-butenyl)-L-ornithine.


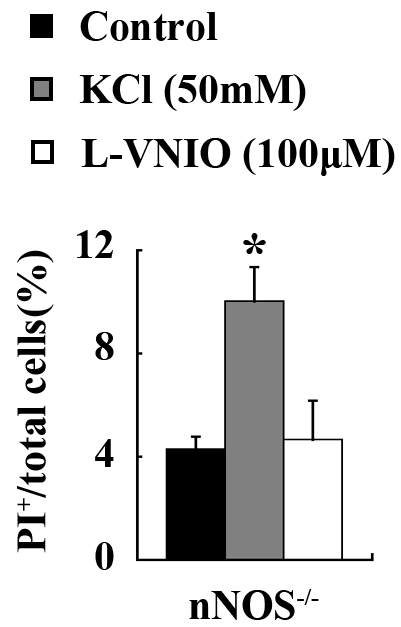


**Supplementary Figure 2.** The effect of KCl and L-VNIO on cell apoptosis when nNOS is knocked out. We treated nNOS^-/-^ cells with 50mM KCl for the first 24 hours or 100μM L-VNIO for the later two days of four-day differentiation. Live cultures were stained with PI which stains dead cells and Hoechst 33342 which stains live and dead cells. Data are means ± SEM (n=3). *, p < .05 as compared with control. Abbreviations: L-VNIO, N5-(1-imino-3-butenyl)-L-ornithine; nNOS^-/-^, gene knockout of neuronal nitric oxide synthase; PI, propidium iodide.

**
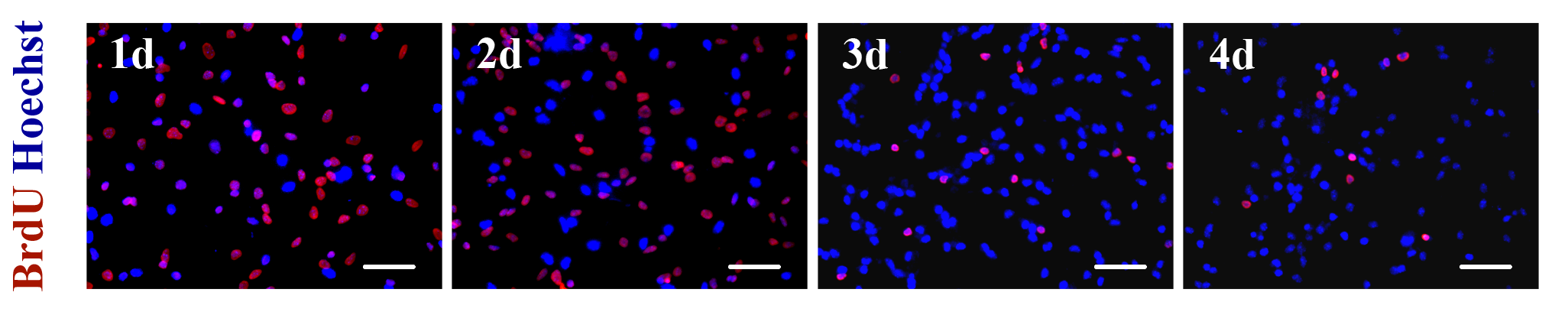
**

**Supplementary Figure 3.** Cell proliferation during differentiation assessed by BrdU incorporation. Cells were treated with 2.5μM BrdU for 24 hours on days 0, 1, 2 and 3 after differentiation of monolayer-cultured embryonic NSCs, immediately followed by fixation and staining for BrdU. BrdU-positive cells during the first two days were much more than that during the later two days, and the ratio of BrdU-labeled cells was approximately 90% (day 1), 80% (day 2), 10% (day 3), 10% (day 4). Scale bar, 50μm. Abbreviations: BrdU, bromodeoxyuridine.
